# Systematic review and meta-analysis of the clinical outcomes of ACEI/ARB in East-Asian patients with COVID-19

**DOI:** 10.1371/journal.pone.0280280

**Published:** 2023-01-12

**Authors:** Nancy Xurui Huang, Qi Yuan, Fang Fang, Bryan P. Yan, John E. Sanderson

**Affiliations:** 1 Department of Cardiology, Affiliated Hangzhou First People’s Hospital, Zhejiang University School of Medicine, Hangzhou, China; 2 Research Division, Institute of Mental Health, Singapore, Singapore; 3 Department of Medicine and Therapeutics, The Chinese University of Hong Kong, Hong Kong, China; 4 Beijing AnZhen Hospital, Beijing Capital Medical University, Beijing, China; Ehime University Graduate School of Medicine, JAPAN

## Abstract

**Background:**

SARS-CoV-2 invades human cells and leads to COVID-19 by direct associating with angiotensin converting enzyme 2 (ACE2) receptors, the level of which may be increased by treatment with angiotensin-converting enzyme inhibitors (ACEIs) and/or angiotensin receptor blockers (ARBs). This meta-analysis aimed to explore the impact of ACEI/ARB treatment on the clinical outcomes of patients with COVID-19 infections among population in the East-Asia region.

**Methods:**

We collected clinical data published from January 2000 to May 2022 in the English databases including PubMed, Embase, and the Cochrane Library. Two reviewers independently screened and identified studies that met the prespecified criteria. Review Manager 5.3 software was used to perform the meta-analysis.

**Results:**

A total of 28 articles were included in this analysis. The results showed that patients who were prescribed with ACEI/ARB had a shorter duration of hospital stay [MD = -2.37, 95%CI (-3.59, -1.14), P = 0.000 2] and a lower mortality rate [OR = 0.61, 95% CI (0.52, 0.70), P<0.000 01] than patients who were not on ACEI/ARB. Furthermore, there was no statistically significant difference in disease severity [OR = 0.99, 95% CI (0.83, 1.17), P = 0.90] between individuals receiving ACEI/ARB or not.

**Conclusions:**

This meta-analysis suggested that the use of ACEI/ARB was not associated with adverse clinical outcomes in East-Asian Covid-19 patients and a reduced mortality and shorter duration of hospital stay among East-Asian population (especially for female subjects) was found. Thus, ACEI/ARB should be continued in patients infected by Covid-19.

## Introduction

Coronavirus-infected pneumonia COVID-19 or SARS-CoV-2 (severe acute respiratory syndrome coronavirus 2) [1] has caused a worldwide pandemic with more than 500 million confirmed infections and over 6 million deaths have been reported as of 1st May 2022 [2]. The pandemic has placed overwhelming pressure on the medical systems globally and has dealt a huge blow to world economies.

Similar to SARS (severe acute respiratory syndrome), the pathogenesis of COVID-19 is closely related to angiotensin converting enzyme 2 (ACE2). It was reported that ACE2 is considered the functional receptor for novel SARS-CoV-2 coronavirus entry into host cells, causing lung, blood vessel, immune and other damage of human beings [3]. Angiotensin-converting enzyme inhibitors (ACEIs) and angiotensin receptor blockers (ARBs) are renin-angiotensin aldosterone system (RAAS) inhibitors, which are widely used for patients with hypertension (HT) and cardiovascular diseases (CVDs). There have been concerns that the use of ACEI/ARB might cause a higher ACE/ACE2 expression and increase the risk of COVID-19 infection and mortality, thus resulting in a worse clinical outcome [4].

To date, some studies and meta-analyses have already explored the correlation between ACEI/ARB and COVID-19, yet they are mostly some small observational studies, or are based on the global population, or analyzed data only up to the early of 2021. The results are still controversial. Considering the relative higher expression of ACE2 in the Asian population compared to other populations [5], the question has been raised of what is the real relationship between ACEI/ARB and COVID-19 in the East-Asian population, and whether such relationship will turn out to be as the same as the global one?

In this study, systematic literature search and meta-analysis aimed to determine a potential association between the prescription of ACEI/ARB and the clinical prognosis COVID-19 in the East-Asian population.

## Methods

### Search strategy

This systematic review was conducted following the Preferred Reporting Items for Systematic Reviews and Meta-Analyses (PRISMA) statement [6]. The systematic review protocol was registered via PROSPERO (https://www.crd.york.ac.uk/PROSPERO/; CRD42022322682). The literature search was conducted on the databases of PubMed, Embase, and the Cochrane Library, from January 2000 to May 2022. Using keywords and Medical Subject Headings (MeSH) terms, such as "novel coronavirus pneumonia", "NCP", "2019-nCoV", "COVID-19", "coronavirus disease 2019", "SARS-CoV-2", "angiotensin converting enzyme inhibitors", "angiotensin receptor blockers", "ACEI”, “ARB ". We limited the search condition to human beings. And research published in languages other than English was not included. Search was not limited to any particular diagnosis or reason for prescription of ACEI/ARB.

### Literature inclusion and exclusion criteria

Inclusion criteria: (1) study design: cohort study; (2) study population: East-Asian adult (>18 years) hospitalized patients with confirmed COVID-19 infection, diagnosed by clinical laboratory or imaging results; (3) groups: the clinical prognosis of the experimental group (treated with ACEI/ARBs) were compared with the control group (not treated with ACEI/ARBs); (4) assessment of outcomes: mortality, severe infection of COVID-19 (including admission to intensive care unit (ICU), mechanical ventilation use or progressing to severe pneumonia), as well as the duration of hospital stay. Exclusion criteria: (1) literature with insufficient or incorrect data that cannot be analyzed; (2) manuscripts that did not meet the inclusion criteria; (3) repeated publications; (4) articles with only abstracts.

### Literature screening and data extraction and quality evaluation

In this study, two reviewers (N.H. and Q.Y.) independently carried out literature screening and identified studies that met the prespecified criteria. Any discrepancy was resolved through discussion by the third independent reviewer (F.F.) to reach a consensus. In terms of quality evaluation, two reviewers independently conducted methodological quality evaluation of the included studies according to the Newcastle-Ottawa Scale (NOS) [7], a 9-point scale ranging from 0 (high risk of bias) to 9 (low risk of bias). According to the NOS score, the studies were divided into high quality (> 7 points), medium quality (3–6 points), and low quality (< 3 points).

### Statistical analysis

In this study, Review Manager 5.3 statistical software was used to conduct a meta-analysis of the included literature. Mean Difference (MD) and 95% confidence interval were used for continuous variables, and Odds Ratio (OR) and 95% confidence interval (95% CI) were used for binary variables. If the heterogeneity test P≤0.1 or I^2^>50%, it indicates that the index is statistically different between the studies, and the Random Effects Model (Random) is used to merge. If the heterogeneity test P>0.1 and I^2^ <50%, it indicates that there is no statistical difference in this indicator between studies, and the Fixed Effects Model (Fixed) is used for merging. Publication bias was checked by funnel chart analysis.

## Results

### Literature search results

**Fig 1:**
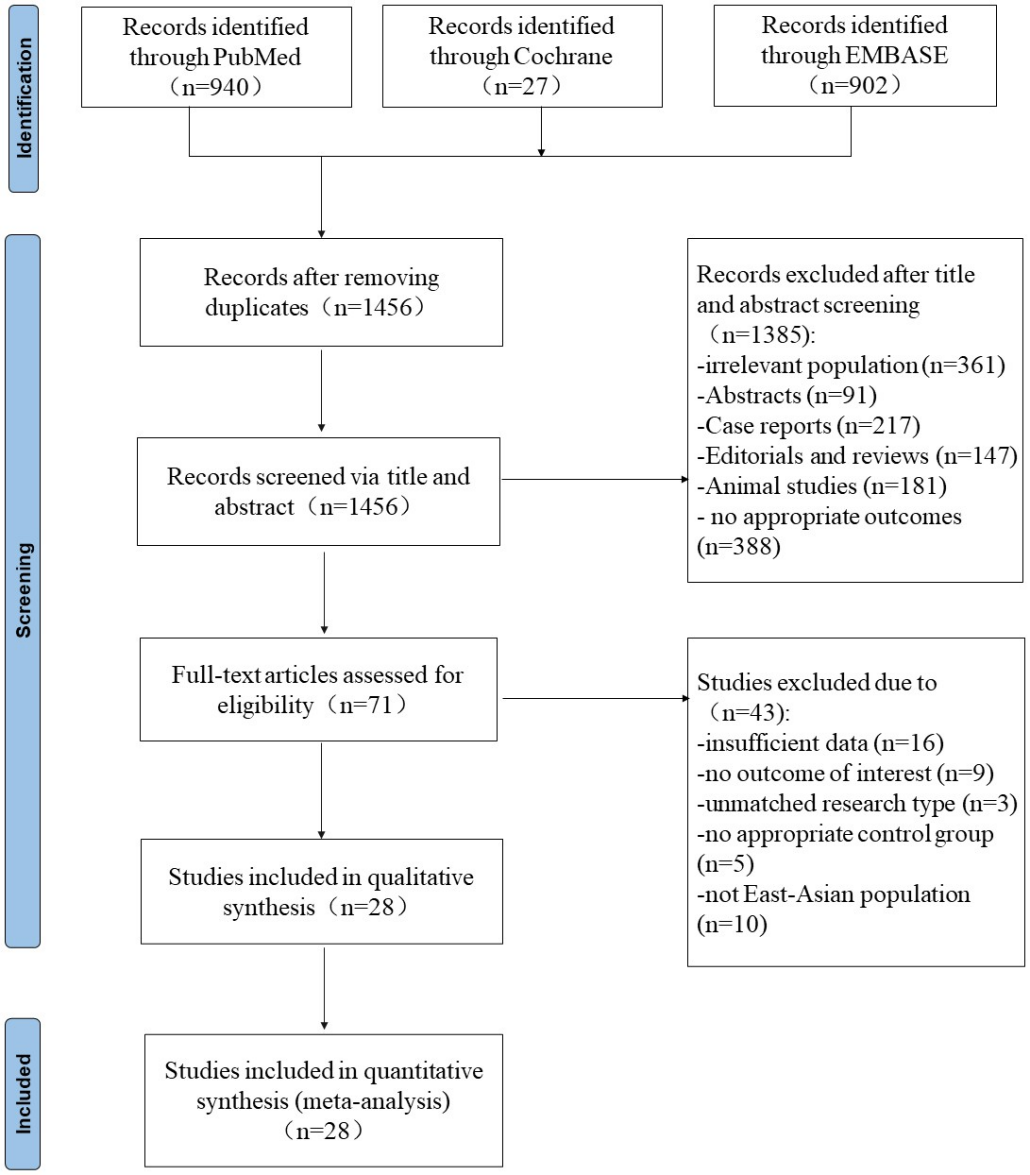
Screening flow chart for the systematic review and metaanalysis. It includes selecting and exclusion reasons which followed Preferred Reporting Items for Systematic Review and Metaanalysis (PRISMA) statement.

A preliminary screening based on the search strategy was conducted and identified 1,869 relevant studies. After removing duplicates, 413 papers were deleted. The remaining 1456 articles were screened by reading the titles and abstracts, and 71 articles were retrieved for full-paper review. Finally, 28 articles met the eligibility criteria mentioned above and were included in our meta-analysis (Fig 1). The included researches are all observational studies.

These included articles evaluated a total of 14,963 East-Asian COVID-19 patients, including 7,533 who were prescribed with ACEI/ARBs and 7,430 who were not. All studies had explored the impact of the use of ACEI/ARBs on the prognosis of East-Asian COVID-19 patients. In terms of the clinical prognosis, 23 studies considered mortality as the indicator; 17 studies used the severity of COVID-19 as the indicator; 9 studies employed the duration of hospital stay as the evaluation indicator. We choose death as the most severe clinical outcome. The basic characteristics and quality evaluation of the included studies are shown in Table 1.

**Table 1:** Basic characteristics of the included studies.

Study	Region	Research type	Population	Number	ACEI/ARB	Non-ACEI/ARB	Clinical Outcome			NOS			
										Selection	Comparability	Outcome	Total
Hu J2020 [13]	China	Prospective cohort study	HT	149	65	84	①	②	③	4	1	1	6
Huang Z2020 [9]	China	Prospective cohort study	HT	50	20	30	①	②	③	4	1	2	7
Tan N-D2020 [8]	China	Retrospective cohort study	HT	100	31	69	①	②	③	4	1	3	8
Zhang Y2020 [10]	China	Retrospective cohort study	HT	75	28	47	①	②	③	4	1	3	8
Pan W2020 [24]	China	Retrospective cohort study	HT	282	41	241		②	③	4	1	3	8
Wang T2021 [11]	China	Retrospective cohort study	HT	93	35	58	①	②	③	4	1	1	6
Wang W2021 [12]	China	Retrospective cohort study	HT	67	22	45	①	②		4	1	2	7
Huang L2021 [23]	China	Retrospective cohort study	Whole ^a^	152	38	114		②	③	4	2	2	8
Gao C2020 [17]	China	Retrospective cohort study	HT	710	183	527		②	③	4	2	3	9
Li J2020 [18]	China	Retrospective cohort study	HT	362	115	247		②	③	4	1	3	8
Li X2020 [19]	China	Retrospective cohort study	Whole ^b^	545	42	503		②		4	1	2	7
Liu Y2020 [20]	China	Retrospective cohort study	HT	78	22	56		②		4	1	2	7
Meng J2020 [21]	China	Retrospective cohort study	HT	42	17	25		②	③	4	1	3	8
Yang G2020 [14]	China	Retrospective cohort study	HT	126	43	83	①	②	③	4	1	3	8
Yuan Y2020 [15]	China	Retrospective cohort study	HT	232	108	124	①			4	2	2	8
Chen Y2020 [16]	China	Retrospective cohort study	HT	71	32	39	①		③	4	1	3	8
Meng X2021 [28]	China	Retrospective cohort study	HT	259	73	186			③	4	1	3	8
Wang HY2021 [29]	China	Retrospective cohort study	HT	590	141	449			③	4	1	3	8
Zhang P2020 [30]	China	Retrospective cohort study	HT	1128	188	940			③	4	2	3	9
Park J2021 [26]	Korea	Retrospective cohort study	HT	1885	1098	787		②		4	1	3	8
Matsuzawa Y2020 [25]	Japan	Retrospective cohort study	HT	39	21	18		②	③	4	1	2	7
Hwang JM2020 [33]	Korea	Retrospective cohort study	Whole ^c^	103	13	90			③	4	1	3	8
Bae S2020 [32]	Korea	Retrospective cohort study	HT or CVDs ^d^	864	359	505			③	4	2	2	8
Kim JH2021 [27]	Korea	Retrospective cohort study	HT	1290	682	608		②	③	4	1	2	7
Lee J2021 [35]	Korea	Retrospective cohort study	HT	1609	1041	568			③	4	1	2	7
Kang SH2021 [34]	Korea	Retrospective cohort study	HT	1044	782	262			③	4	1	1	6
Seo J2021 [36]	Korea	Retrospective cohort study	HT	1644	1217	427			③	4	1	3	8
Bae JH2021 [31]	Korea	Retrospective cohort study	HT	1374	1076	298			③	4	1	2	7

①duration of hospital stay; ②severity; ③mortality.

HT, hypertension; CVDs, cardiovascular diseases.

# a: HT 36%, CVDs 15%; b: HT 30.3%, CVDs 6.2%; c: HT 55.3%, CVDs 12%; d: HT 98.9%, CVDs 33%.

### Meta-analysis results

#### ACEI/ARB usage could shorter duration of hospital stay of COVID-19

**Fig 2:**
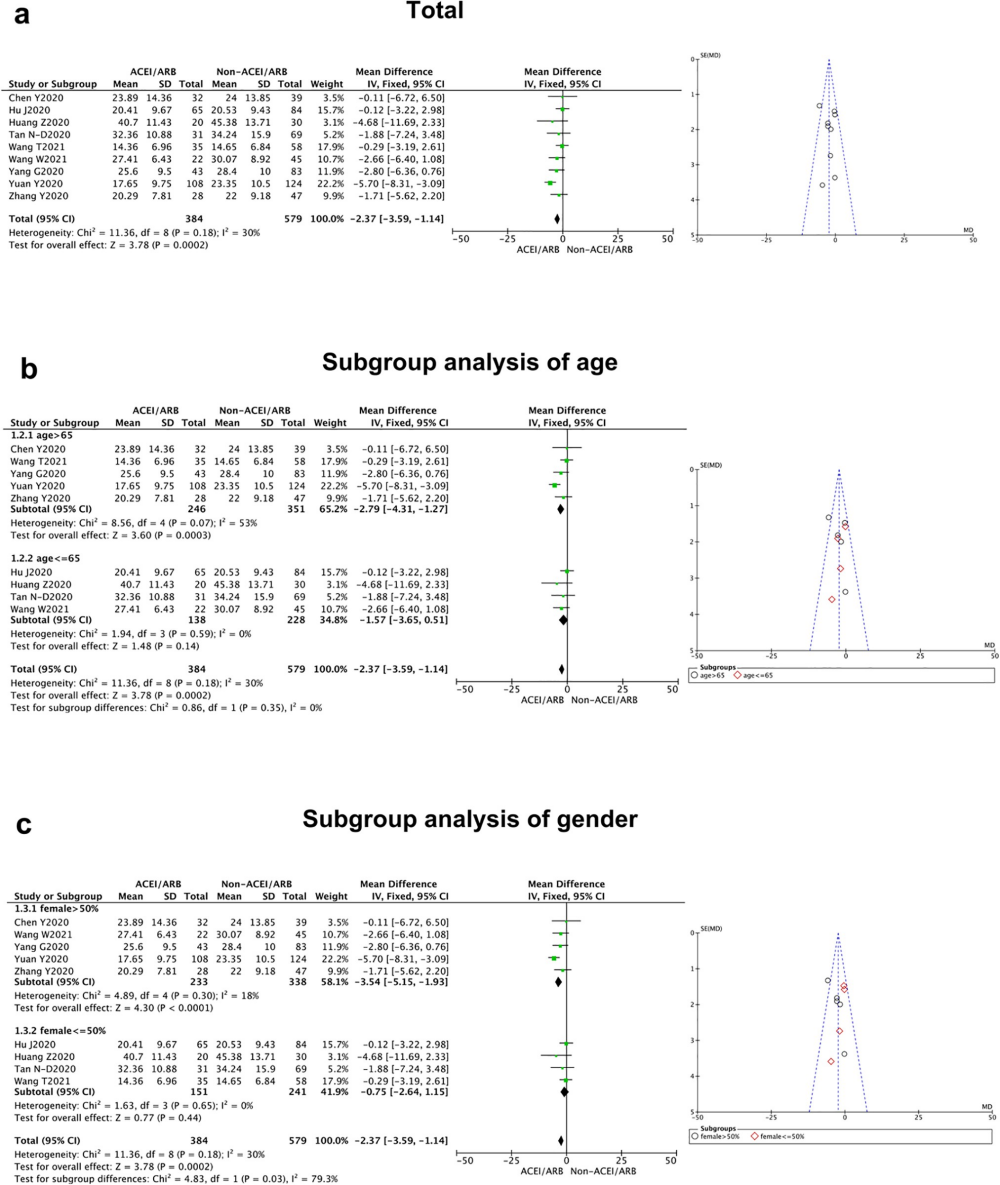
Duration of hospital stay of East-Asian Covid-19 patients treated with ACEI/ARB vs. untreated subjects. The duration of hospital stay data of MD and 95% CI from 9 studies were pooled in this meta-analysis and the result of metaanalysis was described as a forest plot. (a) The whole group analysis; (b)The subgroup analysis of age;(c) The subgroup analysis of gender.

Nine studies estimated the impact of ACEI/ARB treatment on the duration of hospital stay in East-Asian patients with COVID-19 [8–16]. The duration of hospital stay of COVID-19 patients treated with ACEI/ARB was significantly shorter than those who did not receive ACEI/ARB treatment [MD = -2.37, 95% CI(-3.59, -1.14), *P* = 0.000 2], and there was no significant heterogeneity between studies (*P* = 0.18, I^2^ = 30%) (Fig 2, Table 2).

**Table 2:** Clinical outcomes of East-Asian Covid-19 patients treated with ACEI/ARB vs. untreated subjects (all studies).

Outcomes	No. of Studies	Pooled OR/MD (95% CI)	*P* for Heterogeneity	I^2^	*P*	*P* for subgroup differences	Model Used
**Duration of hospital stay**	9 [8–16]	-2.37(-3.59, -1.14)	0.18	30%	0.0002		Fix
(Mean age)						0.35	
>65 y	5	-2.79(-4.31, -1.27)	0.07	53%	0.0003		
< = 65 y	4	-1.57(-3.65, 0.51)	0.59	0%	0.14		
(Female sex)						0.03	
>50%	5	-3.54(-5.15, -1.93)	0.30	18%	<0.000 01		
< = 50%	4	-0.75 (-2.64, 1.15)	0.65	0%	0.44		
**Severity**	17 [8–13, 17–27]	0.99 (0.83,1.17)	0.15	27%	0.90		Fix
(Mean age)						0.21	
>65 y	7	0.85(0.63, 1.14)	0.05	52%	0.27		
< = 65 y	10	1.07 (0.87, 1.32)	0.54	0%	0.53		
(Female sex)						0.20	
>50%	4	1.21(0.85, 1.72)	0.26	25%	0.29		
< = 50%	13	0.93(0.76, 1.13)	0.18	27%	0.46		
**Mortality**	23 [8–11, 13, 16–18, 21–25, 27–36]	0.61 (0.52,0.70)	0.19	20%	<0.000 01		Fix
(Mean age)						0.66	
>65 y	12	0.59(0.49, 0.71)	0.09	38%	<0.000 01		
< = 65 y	11	0.63(0.49, 0.81)	0.48	0%	0.0004		
(Female sex)						0.31	
>50%	6	0.65(0.53, 0.79)	0.34	12%	<0.0001		
< = 50%	17	0.56(0.44, 0.70)	0.15	26%	<0.000 01		

#### ACEI/ARB usage would not further worsen the severity of COVID-19

**Fig 3:**
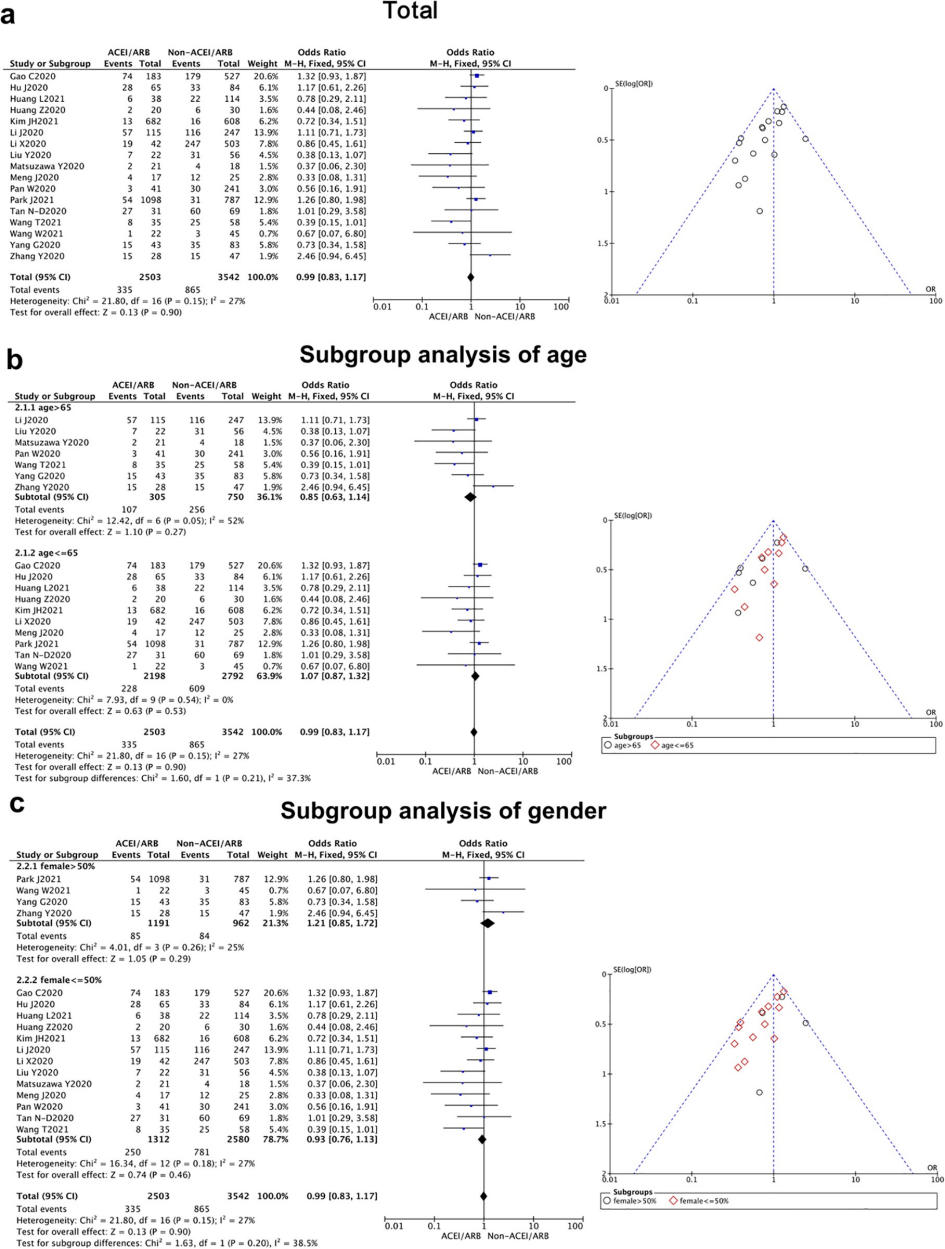
Severity of East-Asian Covid-19 patients treated with ACEI/ARB vs. untreated subjects. The severity data of OR and 95% CI from 17 studies were pooled in this meta-analysis and the result of meta-analysis was described as a forest plot. (a) The whole group analysis; (b) The subgroup analysis of age; (c) The subgroup analysis of gender.

A total of 17 articles compared whether the use of ACEI/ARB affect the severity of COVID-19 [8–13, 17–27]. Our study showed that there was no significant difference in the severity of coronavirus disease between the two groups [OR = 0.99, 95% CI (0.83, 1.17), *P* = 0.90] (Fig 3, Table 2). Our research result indicated that the use of ACEI/ARB will not further worsen the severity of this pneumonia. There was no statistical heterogeneity among the studies (*P* = 0.15, I^2^ = 27%), so a fixed-effect model can be used to combine results. A funnel chart test found that the distribution of the funnel chart was basically symmetrical, showing an inverted funnel shape, suggesting that the publication bias of this part of study was low (Fig 3).

#### ACEI/ARB usage could decrease the mortality of COVID-19

**Fig 4:**
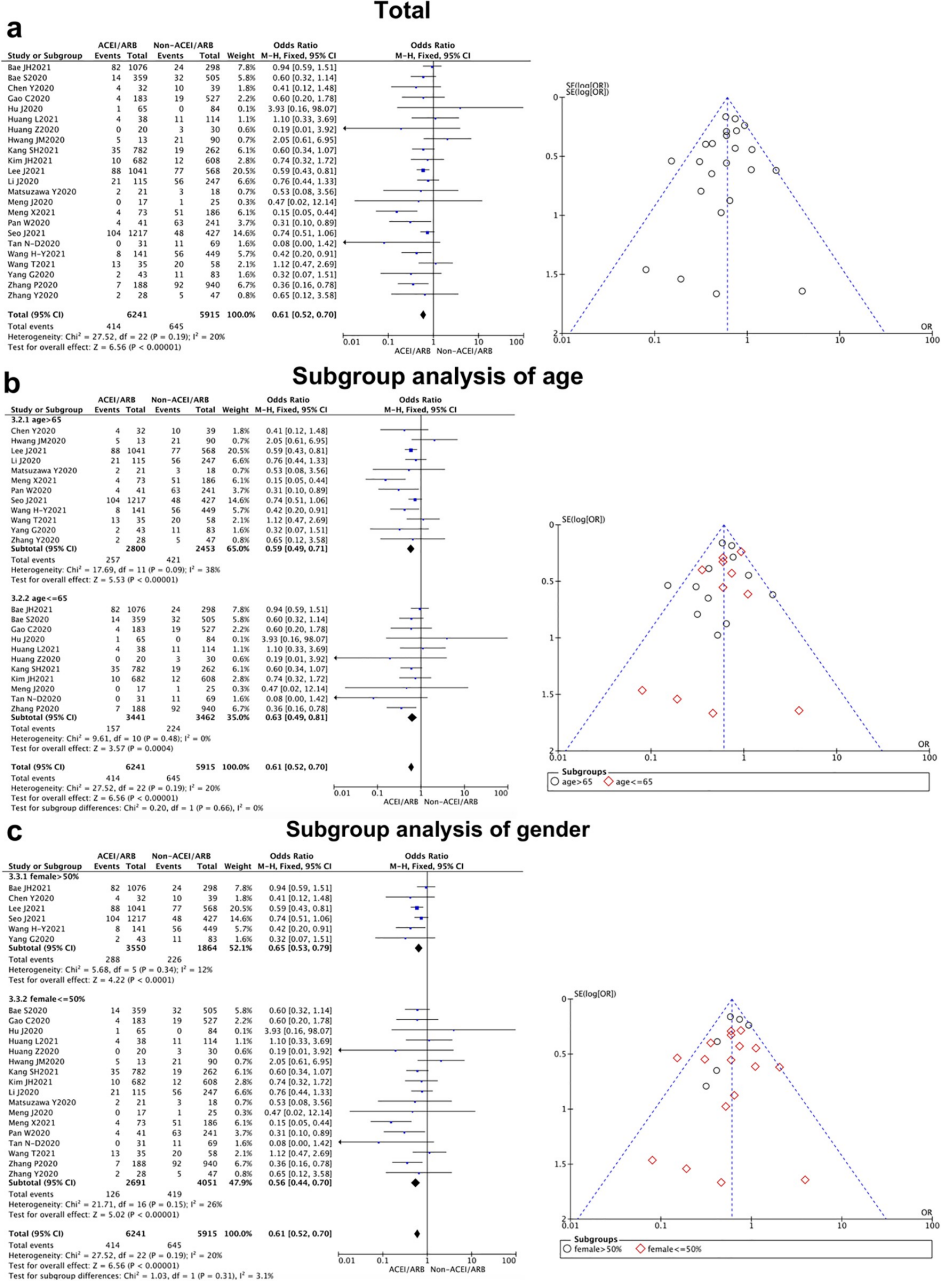
Mortality of East-Asian Covid-19 patients treated with ACEI/ARB vs. untreated subjects. All-cause mortality data of OR and 95% CI from 23 studies were pooled in this meta-analysis and the result of meta-analysis was described as a forest plot. (a) The whole group analysis; (b) The subgroup analysis of age; (c) The subgroup analysis of gender.

Of the literature included in our analysis, 23 articles compared the mortality of COVID-19 patients treated with and without ACEI/ARB [8–11, 13, 16–18, 21–25, 27–36]. The result of analysis pointed out that there was a statistically significant difference in mortality between whether ACEI/ARB were used or not [OR = 0.61, 95% CI (0.52, 0.70), *P*<0.000 01] (Fig 4, Table 2). The mortality of COVID-19 patients who were prescribed with ACEI/ARB was lower than that of patients who were not. The fixed effect model was used to combine studies as no statistical difference was found between groups(*P* = 0.19, I^2^ = 20%). The publication bias analysis of related studies on the impact of ACEI/ARB on the mortality of COVID-19 patients is shown in Fig 4. The scattered points of the funnel chart are concentrated on both sides of the invalid line, in an inverted funnel shape, and the distribution on both sides is basically symmetrical, indicating that there is low publication bias.

#### Was the effect of ACEI/ARB influenced by age or gender?

When we separated the studies by age and gender for subgroup analysis, a significant difference could be found in the duration of hospital stay between the female and male patients (*P* = 0.03), while no statistical difference was revealed from the age subgroups analysis (*P* = 0.35). It showed East-Asian female patients could benefit further from ACEI/ARB prescription with a shorter duration of hospital stay. In addition, we performed the same subgroup analysis in other clinical outcomes (severity and mortality), and no meaningful differences could be detected between the elder and younger patients (*P* = 0.21 for severity, *P* = 0.66 for mortality), as well as female vs. male subgroups (*P* = 0.20 for severity, *P* = 0.31 for mortality) (Figs 2–4).

#### The effect of ACEI/ARB is more obvious in hypertension population

**Fig 5:**
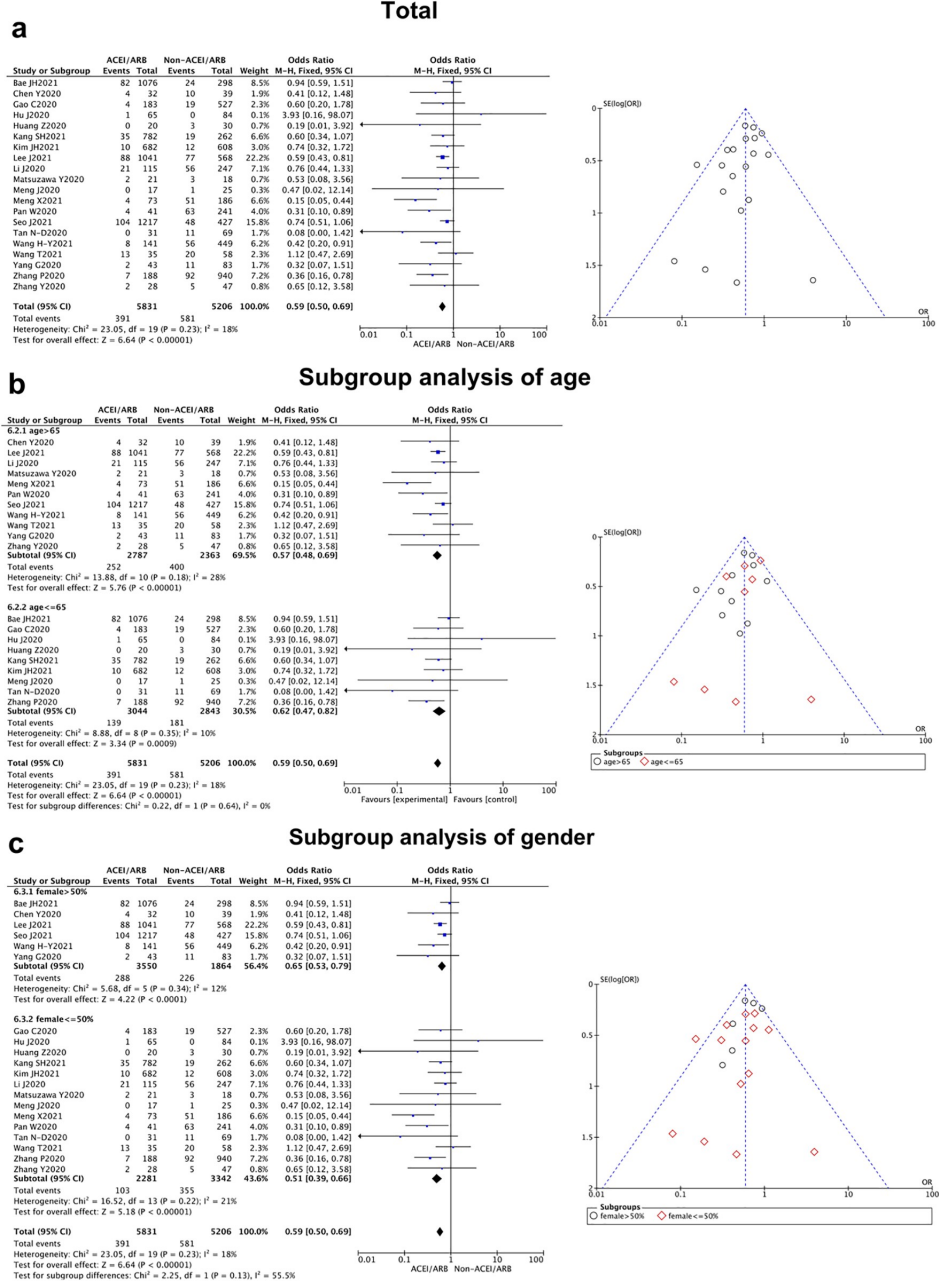
Mortality of East-Asian Covid-19 patients treated with ACEI/ARB vs. untreated subjects (HT subgroup). All-cause mortality data of OR and 95% CI from 20 studies were pooled in this meta-analysis and the result of meta-analysis was described as a forest plot. (a) The whole group analysis; (b) The subgroup analysis of age; (c) The subgroup analysis of gender.

**Fig 6:**
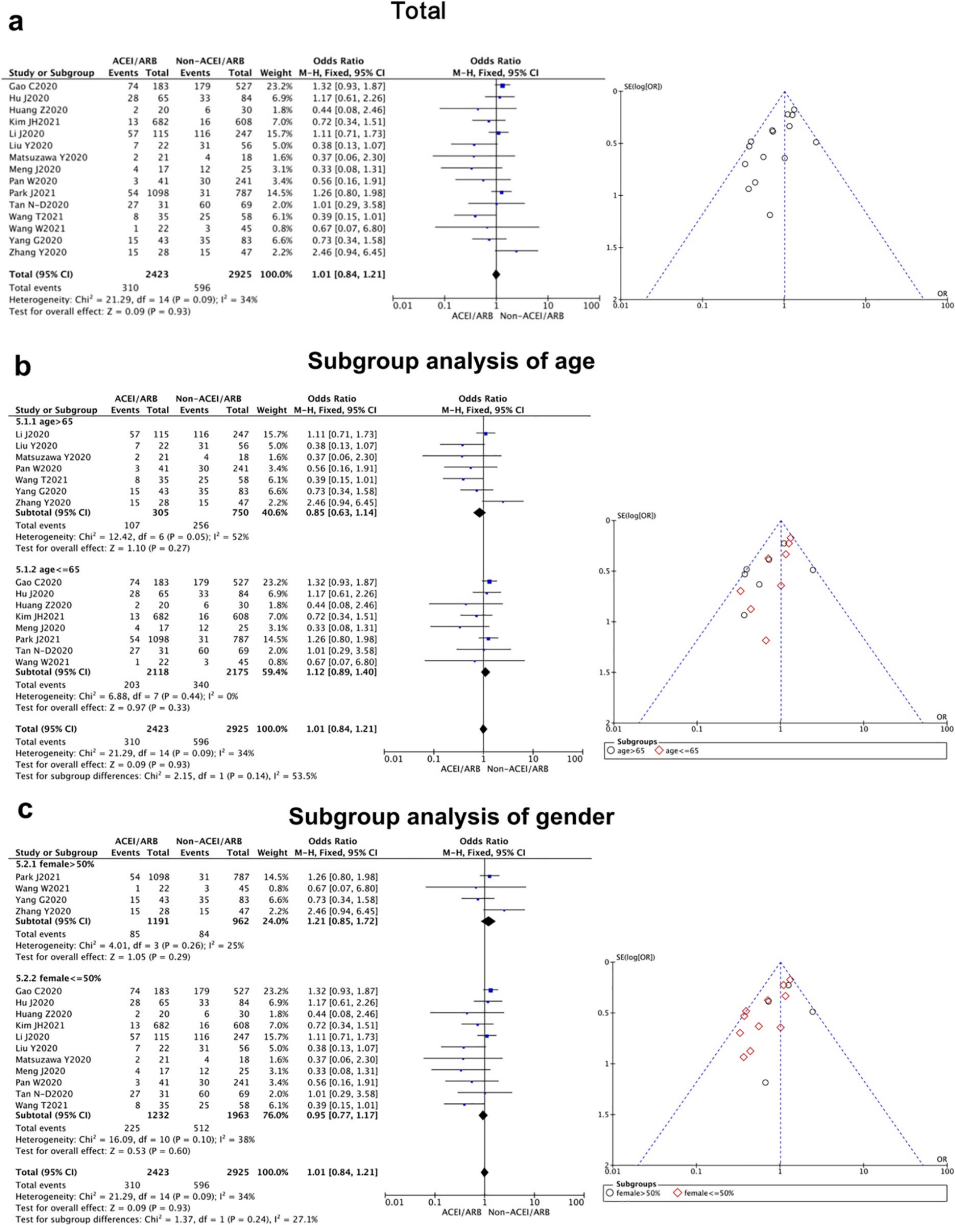
Severity of East-Asian Covid-19 patients treated with ACEI/ARB vs. untreated subjects (HT subgroup). The severity data of OR and 95% CI from 15 studies were pooled in this meta-analysis and the result of meta-analysis was described as a forest plot. (a) The whole group analysis; (b) The subgroup analysis of age; (c) The subgroup analysis of gender.

ACEI/ARB is one of the most widely used anti-hypertensive medications. 24 of the included studies were on hypertension patients and the remaining 4 studies included other patients. We had also conducted subgroup analysis to explore the impact of ACEI/ARB among hypertension patients. The subgroup analysis revealed a more obvious reductions in mortality among patients in the hypertension subgroup who were receiving ACEI/ARB [OR = 0.59, 95% CI (0.50, 0.69), *P*<0.000 01] (Fig 5, Table 3). Additionally, for the outcome of severity in the hypertension subgroup, the result indicated a non-statistically significant difference between whether ACEI/ARB was used or not (Fig 6, Table 3). Analysis of mortality in the CVDs subgroup showed a similar fall in mortality but it was not significant (S1 Table).

**Table 3:** Clinical outcomes of East-Asian Covid-19 patients treated with ACEI/ARB vs. untreated subjects (HT subgroup).

Outcomes	No. of Studies	Pooled OR (95% CI)	*P* for Heterogeneity	I^2^	*P*	*P* for subgroup differences	Model Used
**Severity**	15 [8–13, 17, 18, 20–22, 24–27]	1.01 (0.84,1.21)	0.09	34%	0.93		Fix
(Mean age)						0.17	
>65 y	7	0.85(0.63, 1.14)	0.05	52%	0.27		
< = 65 y	8	1.12 (0.89, 1.40)	0.44	0%	0.33		
(Female sex)						0.24	
>50%	4	1.21(0.85, 1.72)	0.26	25%	0.29		
< = 50%	11	0.95(0.77, 1.17)	0.10	38%	0.60		
**Mortality**	20 [8–11, 13, 16–18, 21, 22, 24, 25, 27–31, 34–36]	0.59 (0.50,0.69)	0.23	18%	<0.000 01		Fix
(Mean age)						0.64	
>65 y	11	0.57(0.48, 0.69)	0.18	28%	<0.000 01		
< = 65 y	9	0.62(0.47, 0.82)	0.35	10%	0.0009		
(Female sex)						0.13	
>50%	6	0.65(0.53, 0.79)	0.34	12%	<0.0001		
< = 50%	14	0.51(0.39, 0.66)	0.22	21%	<0.000 01		

## Discussion

This meta-analysis systematically evaluated the impact of ACEI/ARB on the clinical prognosis of COVID-19 patients in East-Asia. The result of the study showed that ACEI/ARB would not aggravate the severity of a new COVID-19 infection in East-Asian patients, nor would it prolong the duration of hospital stay of East-Asian patients with COVID-19, and that the use of ACEI/ARB can reduce the risk of death to a certain extent, especially those with hypertension.

Further, the results of this study have potentially important implications for clinical practice. Recently, there has been controversy regarding the use of RAAS system inhibitors during the COVID-19 pandemic. On the one hand, some experts believed that the application of ACEI/ARB would accelerate the spread of COVID-19 and increase the severity of new COVID-19 induced pneumonia, so the drugs should be stopped [37]. On the other hand, some researchers believed that RAAS system inhibitors would not only not worsen the clinical prognosis of COVID-19, but also reduce the mortality rate, so they should continue to be used [38].

Multiple studies have shown that individuals with hypertension and other cardiovascular diseases are more likely to be infected with COVID-19, and further had the worse clinical outcomes [39]. Research has demonstrated that the pathophysiology of COVID-19 is similar to SARS: the novel coronavirus enters cell by binding to the ACE2 receptor of the host-cell. The ACE2 receptor is highly expressed in the human body, such as in lung tissues, the digestive system, vascular endothelial cells and vascular smooth muscle cells. The spike protein of SARS-CoV-2 mainly binds to ACE2 in human’s respiratory and lung tissues as a cellular receptor, leading to a series of pathological changes and causing damage to the respiratory system. ACEI/ARB are widely used in the treatment of hypertension and cardiovascular diseases. Animal experiments have shown that ACEI/ARB could inhibit ACE while increasing ACE2 receptors [40]. Thus, some experts hypothesize that these medications may increase the susceptibility to viruses by increasing the expression of ACE2 receptor, thereby speeding up the spread of COVID-19 [41]. However, our results do not support this statement. Although some studies have reported that RAAS system inhibitors may increase the expression of ACE2 receptors, the results of these tests are not consistent. Most of them are animal tests, and a small number of human tests are studies with small sample sizes. Animal studies about the effect of ACEI/ARB on ACE2 are mostly focused on heart and kidney tissues [42]. And human studies mainly detect circulating ACE2 or soluble ACE2 receptor levels in the urine or plasma, and these indicators may not truly reflect the ACE2 levels in other tissues and organs [43, 44]. In 2017, a human study by Masato et al. evaluated the expression of Urinary and Plasma ACE2 receptors in 605 patients who used RAAS system inhibitors. They found the use of RAAS system inhibitors is not an independent predictor of the Urinary and Plasma ACE2 levels, and their levels could also be influenced by other factors like high blood pressure, liver dysfunction and so on [45]. We know that SARS-CoV-2 enters the human body mainly through the ACE2 receptor of respiratory tract. The concentration of medication in different organs and tissues is different, and the expression and distribution of enzymes and receptors in different organs and tissues are significantly different as well. Thus, ACEI/ARB may not have the similar effect in lung tissue as in other tissues or organs. Additionally, a recent study finds that ACE2 levels are not increased in the respiratory tract in patients taking long term ACEI and ARBs [46]. But further studies are required to confirm this finding and its relevance to the action of ACEI/ARBs in reducing mortality. As noted above many other organs and systems are potentially involved and outcome may be determined more by these than the infection in the lung per se.

It has pointed out that the current evidence does not support the harmful effects of RAAS system inhibitors on COVID-19 infection, and it is recommended not to discontinue RAAS system inhibitors [47]. In fact, the evidence of its benefits is substantial. Our meta-analysis also confirmed this opinion. Some researchers even advocate the continued use of RAAS system inhibitors, because the RAAS system is a key target for the treatment of acute lung injury [48]. ACE2 is not only the receptor to help SARS-CoV-2 entering into cell but also will be down-regulate by this virus, subsequently reducing the increased level of angiotensin II which may play a great role of organ injury in COVID-19. Nevertheless, ACEI/ARBs could inhibit the activity of angiotensin II, as well as increase the expression of ACE2 to offset the reduction of ACE2 in lung tissue caused by the virus, thereby play a protective role [49]. More notably, some previous researchers have demonstrated in patients with pneumonia and acute respiratory distress syndrome that ACEI/ARB treatment could improve clinical outcomes [50]. Recently, some studies also found ACEI/ARB can significantly reduce COVID-19 caused pulmonary inflammation by inhibiting the levels of IL-6, C-reactive protein and calcitonin [14, 21].

At the same time, RAAS system inhibitors have been proven to be effective in protecting the cardiovascular and renal system [51]. Discontinuation of ACEI/ARB will increase the chance of decompensation in high-risk patients, because the specific benefits of ACEI/ARB in slowing down ventricular remodeling cannot be replaced by other drugs. It is in consistent with our meta-analysis result that the CVDs subgroup prescribed with ACEI/ARB showed an observable decline in mortality. Although such decline was not significant, probably because of small numbers. And this question can be explored by future research. In addition, the proper prescription for hypertension requires frequent adjustment of dosages and good management of adverse reactions. Changing prescription would increase the times of hospital visiting, thereby increasing the patients’ exposure time, and finally raising the risk of COVID-19 infection.

Currently, as the sample size of clinical studies on COVID-19 is not large enough, the results of meta-analysis would more truly reflect the reality through combining multiple clinical trials, adjusting weight and model according to the research design, sample size and results. The main advantages of our meta-analysis are large samples and comprehensive searches to provide more accurate and reliable analyses.

In addition, the meta-analyses reported so far have mostly been multiracial analysis; there are few studies targeting the East-Asian population alone. Different races have different numbers of ACE2 receptors, which leads to different responses to medication. Studies have shown that the expression of ACE2 is relatively high among Asian women and young people [5]. Therefore, a meta-analysis specifically targeted at the East-Asian population is particularly important for the guidance of prescription of ACEI/ARB.

Additionally, we investigated whether age and gender might be relevant, ACE2 receptor density is comparatively high in Asian women and a younger population. Our meta-analysis of clinical studies finding was consistent with the result revealed from the basic studies at the molecular level that Asian females with higher ACE2 expression have the better clinical outcome [5]. Based on that, gender may be the possible reason that influences the clinical outcome, especially in duration of hospital stay. However, we could not find any statistical difference in severity and mortality between subgroups of elder vs. younger and female vs. male patients. It illustrated that East-Asian COVID-19 patients prescribed with ACEI/ARB had lower mortality, regardless of whether they were elder or younger, female or male.

However, this meta-analysis still has some limitations. Firstly, the included articles are all observational. Observational studies could only reflect correlations but cannot clarify the causal relationship. Therefore, it is impossible to confirm the causal relationship between ACEI/ARB treatment and the clinical prognosis of COVID-19 patients. Further research, such as large randomized controlled clinical trials, are still needed to confirm our results. Second, most studies lack specific data of RAAS inhibitor dosage, and different dosages may cause different results, which may affect our results. Third, as most of the included literature is retrospective studies, recall bias is inevitable, which may affect the reliability of the conclusions.

## Conclusions

Currently, millions of patients in the world have used ACEI/ARB, and the COVID-19 epidemic is still raging, especially due to the recent outbreak of novel coronaviruses of Delta and Omicron. Therefore, there is an urgent need for medication guidance for ACEI/ARB. Our result shows that for East-Asian COVID-19 patients, the use of ACEI/ARB will not increase the severity of COVID-19, and the use of ACEI/ARB can significantly reduce duration of hospital stay (especially for female population) and all-cause mortality of COVID-19 patients. These results indicate that COVID-19 patients should continue to receive treatment with ACEI/ARB, especially among the patients who are currently using ACEI/ARB. However, it still needs further animal experiments and large prospective studies to confirm these results, and to explore the possible protective mechanism of ACEI/ARB.

The results of our research provide new additional information to develop guidelines for the use of ACEI/ARB treatment during the ongoing COVID-19 epidemic, and may help to control the spread of the virus and improve the clinical outcome of COVID-19 patients.

## Supporting information

S1 Table Clinical outcomes of East-Asian Covid-19 patients treated with ACEI/ARB vs. untreated subjects (CVDs subgroup). (DOCX)

S1 Checklist PRISMA 2020 checklist. (DOCX)
