# Supplementary material for: Systematic review and meta-analysis of the clinical outcomes of ACEI/ARB in East-Asian patients with COVID-19
Source: PLoS One. 2023 Jan 12;18(1):e0280280. doi: 10.1371/journal.pone.0280280 (PMC9836310; doi:10.1371/journal.pone.0280280)
Supplement: S1 Table — (DOCX) [file pone.0280280.s001.docx]

**Supplementary material**

**Supplemental Table S1. Clinical outcomes of East-Asian Covid-19 patients treated with ACEI/ARB vs. untreated subjects ( CVDs subgroup)**

**Supplemental Table S1. Clinical outcomes of East-Asian Covid-19 patients treated with ACEI/ARB vs. untreated subjects (CVDs subgroup)**

| **Outcomes** | **No. of Studies** | **Pooled OR**  **（95% CI）** | ***P* for Heterogeneity** | **I^2^** | ***P*** | ***P* for subgroup differences** | | **Model Used** |
| --- | --- | --- | --- | --- | --- | --- | --- | --- |
| **Mortality** | 3 [23, 32, 33] | 0.81 (0.49,1.35) | 0.19 | 40% | 0.43 | |  | Fix |
| （CVDs） |  |  |  |  |  | | 0.10 |  |
| >20% | 1 | 0.60(0.32, 1.14) |  |  | 0.12 | |  |  |
| <=20% | 2 | 1.48(0.63, 3.46) | 0.48 | 0% | 0.36 | |  |  |
